# A protective AAV vaccine for SARS-CoV-2

**DOI:** 10.1038/s41392-022-01158-w

**Published:** 2022-09-05

**Authors:** Simeng Zhao, Junzi Ke, Boyu Yang, Fangzhi Tan, Jie Yang, Chao-Po Lin, Haopeng Wang, Guisheng Zhong

**Affiliations:** 1 iHuman Institute, ShanghaiTech University, 201210 Shanghai, China; 2 School of Life Science and Technology, ShanghaiTech University, 201210 Shanghai, China; 3 Shanghai Clinical Research and Trial Center, Shanghai, China

**Keywords:** Gene delivery, Vaccines

**Dear Editor**,

Severe acute respiratory syndrome coronavirus-2 (SARS-CoV-2) has caused the COVID-19 pandemic, with more than 528 million infections and 6.2 million deaths. To fight against this rapidly spreading pandemic, prophylactic vaccines have been developed using different techniques, such as inactivated virus, messenger RNAs, recombinant proteins, and viral-vectored vaccines. However, rapidly spreading variants of SARS-CoV-2, such as alpha, beta, delta, and omicron variants, have been emerging. Mutations in the spike (S) protein have raised deep concerns for vaccine efficacy since the S protein is the main target of vaccines and neutralizing antibodies. Studies have shown that the efficiency of first-generation vaccines against newly occurred variants were significantly reduced, especially the omicron variant.^1^ Developing new vaccines against current and emerging SARS-CoV2 circulating variants are urgently needed.

Adeno-associated viruses (AAVs) belong to a class of non-enveloped single-stranded DNA dependoparvovirus, and have been widely used as gene delivery vectors because of their efficacy and low immunogenicity.^2^ To date, three AAV-based gene therapies have been approved, including Luxturna (inherited retinal diseases, IRD), Zolgensma (spinal muscular atrophy, SMA), and Glybera (familial lipoprotein lipase deficiency, LPLD). AAVs were also proven to be promising vaccine vectors. Based on our engineered AAV variant, AAV-ie,^3^ we initiated the development of AAV-vectored COVID-19 vaccine candidates, and explored the potential of this AAV as a vaccine platform.

Our earlier study showed that AAV-ie effectively infects many types of cochlear cells, likely due to the insertion of the membrane-permeable peptide.^3^ We hypothesized that AAV-ie might have a broad tropism, including muscles, the main targeted infection sites for vaccines. We first prepared an AAV-ie vector expressing nuclear located GFP reporter (AAV-ie-GFP) and investigated the tissue tropism of AAV-ie by intramuscular (i.m.) or intravenous (i.v.) injecting AAV-ie-GFP at the dose of 1 × 10^11^ genome copies (GCs) per mouse (1 × 10^12^ GC/mL, 100 μL). Tissue-imaging results showed that intramuscular (i.m.) AAV-ie injection resulted in highly transduced muscle cells around the injecting site. In contrast, almost no GFP expression was detected in other organs (Supplementary Fig. S1a), suggesting that i.m. AAV-ie injection resulted in a highly effective and specific expression of targeted proteins in muscles. Intravenous (i.v.) injection resulted in a high level of expression of GFP in livers and hearts. Low extent infection was also observed in spleens (Supplementary Fig. S1b). Besides, neither i.v. nor i.m. injection of AAV-ie resulted in body weight loss (Supplementary Fig. S1c), and hematoxylin-eosin (HE) staining showed that AAV-ie vector delivery did not induce tissue damages (Supplementary Fig. S1d). These results indicated that AAV-ie is an efficient and safe vector for SARS-CoV-2 antigen delivery in vivo.

We then generated a series of vaccine candidates expressing different SARS-CoV-2 S domains with an IL-2 leading signal (Supplementary Fig. S2a). To mimic the trimerized natural S protein, in some cases, a C-terminal T4 fibritin domain (Fd) was fused to stabilize trimer formation. All candidates induced antigen expression in HEK293T cells (Supplementary Fig. S2b). We then vaccinated the mice with the candidates at the dose of 1 × 10^11^ GCs per mouse by i.v. injection and monitored the S-binding antibody titers. Injection of AAV-ie-S1 induced the highest IgG level (Supplementary Fig. S2c, d). Besides, results showed that NTD-Fd, and RBD-Fd induced higher S-binding antibody titers than the monomeric NTD and RBD (Supplementary Fig. S2c, d), these observations were in agreement with the conclusion from previous study that polymers could induce stronger immune response than monomers.^4^ The IgG2a/IgG1 ratios of RBD-trimer, S1, and spike vaccinated sera were also determined, and IgG2a/IgG1 ratio of AAV-ie-S1 was determined as 0.90 (Supplementary Fig. S3), indicating a balanced Th1/Th2 immune response induced by AAV-ie-S1. Thus, S1 was chosen as the immunogen for further investigations.

We vaccinated the mice with AAV-ie-S1 by i.m. injection at the dose of 6 × 10^10^ GCs per mouse and evaluated the humoral responses by analyzing the populations of germinal center B (GcB) and follicular helper T (Tfh) cells, as well as the antigen-specific memory B cells. After vaccination, mice were sacrificed at day 7, and splenic cells were proceeded for GcB and Tfh cell analysis. Results showed that AAV-ie-S1 induces robust GcB (SupplementaryFig. S4a, b) and Tfh (Supplementary Fig. S4c, d) differentiation. Besides, a population of RBD-specific memory B cells could be detected at day 14 in the spleen of AAV-ie-S1 vaccinated mice (Supplementary Fig. S4e, f). These data indicated that AAV-ie-S1 induced robust humoral responses. Further antibody titer monitoring experiments showed that AAV-ie-S1 induces durable responses, with the S-binding geometric mean titers (GMTs) as 3.43 × 10^4^, 3.21 × 10^5^, 1.65 × 10^6^, 3.28 × 10^4^, 4.41 × 10^4^, 9.61 × 10^4^, and 3.72 × 10^4^ on week 4, 6, 8, 12, 16, 20, and 52, respectively (Supplementary Fig. S5a, b). The RBD-binding antibody level was evaluated and the GMTs were determined as 1.82 × 10^4^, 9.93 × 10^4^, 1.36 × 10^5^, 1.26 × 10^5^, 1.10 × 10^5^, 8.75 × 10^4^, 9.55 × 10^4^, and 1.36 × 10^5^ on week 4, 6, 8, 12, 16, 20, and 52, respectively (Supplementary Fig. S5c, d). The immune sera were then tested for their pseudo-virus neutralizing activity, and the neutralizing GMT reached the peak value at week 8 (EC_50_ = 390) then stably maintained (Supplementary Fig. S6). Moreover, AAV-ie-S1 was tested for its thermostability by vaccinating the mice with the vaccine stored at different temperatures. Following antibody titer detection showed that AAV-ie-S1 stimulated a similar immune response after being stored for 2 weeks at 4 °C or the room temperature (Supplementary Fig. S7).

To evaluate the potential of AAV-ie-S1 as a vaccine against SARS-CoV-2, we tested its immunogenicity in the *Macaca fascicularis*. Four male macaques aging 2 to 5 year-old were divided into two groups and treated with AAV-ie-S1 or control AAV (AAV-ie-GFP) by i.m. injection at the dose of 1 × 10^13^ GCs per monkey. High S-binding antibody levels were observed in both macaques on week 4 after vaccination and were steadily maintained in the following 2 months (Fig. 1a–c). The RBD-binding antibody titers were tested, and similar results were observed (Supplementary Fig. S8). The pseudo-virus neutralizing EC_50_ values of monkey 1 immune sera were determined as 236, 482, and 337 on week 4, 8, and 12, respectively; the neutralizing EC_50_ values of monkey 2 immune sera were determined as 1017, 1040, and 638 on week 4, 8, and 12, respectively (Fig. 1d–f). These results showed that AAV-ie-S1 could induce robust humoral responses in non-human primates (NHPs). We then collected peripheral blood mononuclear cells (PBMCs) from monkeys before (week 0) and after (week 8) vaccination to evaluate T cellular responses by stimulating the PBMCs with an S peptide pool. Compared to week 0, the IFNγ^+^, IL-2^+^, and TNFα^+^ populations of CD4^+^ and CD8^+^ T cells were upregulated in week 8 PBMCs (Fig. 1g, h and Supplementary Fig. S9). These results indicated that AAV-ie-S1 induced S-specific T-cell responses after vaccination.

**Fig. 1:**
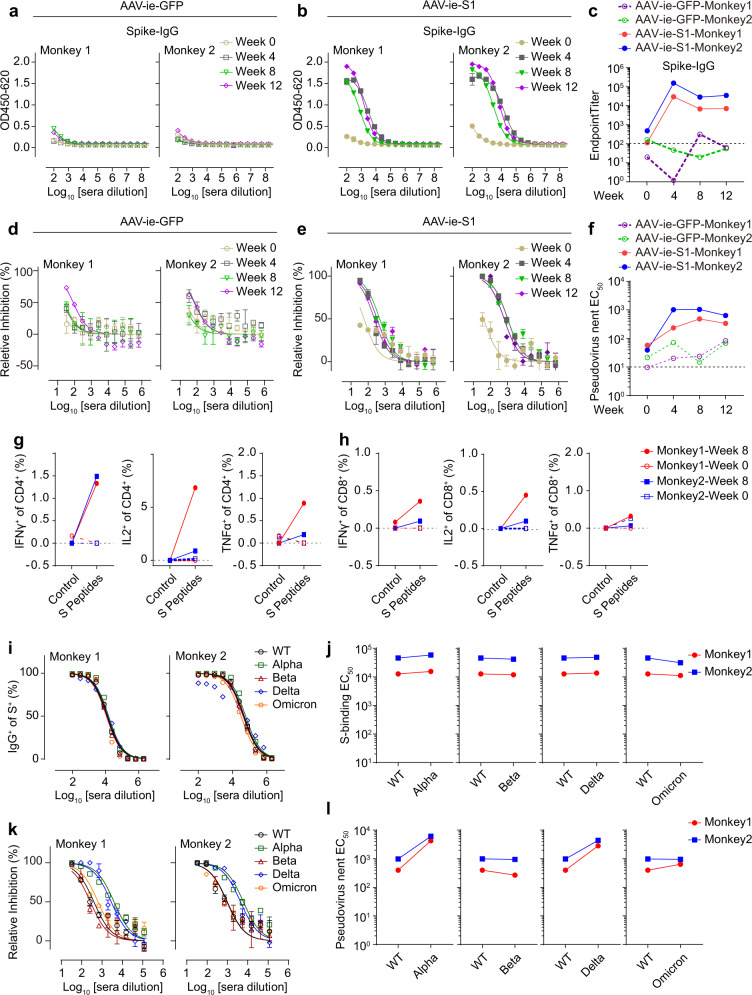
AAV-ie-S1 induces protective immune responses against SARS-CoV-2 in NHPs. **a**–**c** S-binding IgG antibody titers of monkey sera after vaccination with AAV-ie-GFP (**a**) and AAV-ie-S1 (**b**) at indicated time. Endpoint titers were summarized in **c**. Experiments were performed in triplicates. **d**–**f** SARS-CoV-2 pseudo-virus neutralizing activities of monkey immune sera vaccinated with AAV-ie-GFP (**d**) and AAV-ie-S1 (**e**) at indicated time. The neutralizing EC_50_ values were summarized in **f**. Experiments were performed in triplicates. **g**, **h** Populations of CD4^+^ (**g**) and CD8^+^ (**h**) T cells responding to SARS-CoV-2 S peptides before (week 0) and after (week 8) vaccination using intracellular staining of indicated cytokines. **i**, **j** Binding ability of immune sera on WT or mutated S proteins using FACS. Dose curve of IgG^+^ populations under serially diluted immune sera staining (**i**) and S-binding EC_50_ values summary of immune sera (**j**). **k**, **l** Pseudo-virus neutralizing activities of monkey immune sera against WT SARS-CoV-2 and circulating variants. Dose curves were shown in (**k**) and the neutralizing EC_50_ values were summarized in **l**. Experiments were performed in triplicates

A series of SARS-CoV-2 variants are rapidly spreading and some of them, especially omicron related variants, have been validated to be resistant to first-generation vaccines mainly based on full-length spike or RBD domain. To test whether vaccines using S1 domain could avoid immune escape, we evaluated the neutralizing efficiency of AAV-ie-S1 against alpha, beta, delta, and omicron strains. The FACS-based S-binding assay was used to detect the binding abilities of immune sera of NHPs (week 12) to S proteins of wild-type (WT) and mutated SARS-CoV-2 strains (Supplementary Fig. S10). The S-binding EC_50_ values of monkey 1 immune serum to WT, alpha, beta, delta, and omicron proteins were determined as 1.26 × 10^4^, 1.54 × 10^4^, 1.18 × 10^4^, 1.37 × 10^4^, and 1.11 × 10^4^, respectively; and the EC_50_ values of monkey 2 immune serum were 4.56 × 10^4^, 5.83 × 10^4^, 4.15 × 10^4^, 4.84 × 10^4^, and 3.12 × 10^4^, respectively (Fig. 1i, j). These results showed that AAV-ie-S1 immune sera effectively bind to S proteins of WT SARS-CoV-2, alpha, beta, delta and omicron variants. We also tested the binding abilities of mice immune sera, similar to NHPs, the binding activities of mice sera were not significantly affected by the mutations (Supplementary Fig. S11a). The pseudo-virus neutralizing experiments were then carried out to determine the potential efficacy of AAV-ie-S1 immune sera to neutralize alpha, beta, delta, and omicron variants of SARS-CoV-2. Intriguingly, compared to WT, the neutralizing abilities of NHPs immune sera against beta and omicron strains, which have been reported to be resistant to first-generation vaccines, were not significantly decreased (EC_50_ values against beta strain: 397 v.s. 267 for monkey 1; 985 v.s. 943 for monkey 2; EC_50_ values against omicron strain: 397 v.s. 637 for monkey 1; 985 v.s. 950 for monkey 2) (Fig. 1k, l). Interestingly, the neutralizing activities against alpha and delta strains were increased (EC_50_ values against alpha strain: 397 v.s. 4261 for monkey 1; 985 v.s. 6104 for monkey 2; EC_50_ values against delta strain: 397 v.s. 2830 for monkey 1; 985 v.s. 4398 for monkey 2) (Fig. 1k, l). We also tested the neutralizing efficiency of mice immune sera to these circulating variants, and no significant immune escape was observed (Supplementary Fig. S11b). Meanwhile, the neutralizing activity against alpha strain was 1.8 folds increased (Supplementary Fig. S11b). These observations were consistent with the results of NHPs immune sera. Collectively, these results support that AAV-ie-S1 vaccine holds the potential to fight against the emerging SARS-CoV-2 variants.

In summary, we developed a new type of AAV-vectored SARS-CoV-2 vaccine, AAV-ie-S1. AAV-ie-S1 vaccine showed several advantages, such as thermostability, high efficiency, safety, and single-dose vaccination. AAV-ie-S1 induces robust Tfh and GcB cell differentiation, as well as memory B-cell responses. Furthermore, the IgG2/IgG1 ratio of AAV-ie-S1 immune sera indicated that AAV-ie-S1 could induce a balanced Th1/Th2 immune response. Cellular response assays also showed that AAV-ie-S1 induces S-specific Th1 responses. These results suggested that AAV-ie-S1 could avoid biased Th2 response, which is responsible for vaccine-associated enhanced diseases. More importantly, our data showed AAV-ie-S1 vaccinated sera avoided mutational immune escape and neutralized emerging variants efficiently. Besides, a recently published study describing another AAVrh32.33-vectored SARS-CoV-2 vaccine also showed that AAV-vectored full-length spike induced robust humoral and cellular responses and protected against SARS-CoV-2 challenge in vivo.^5^ This work, together with our present work, indicate the potential and advantages of AAV-vectored vaccines, and this strategy may provide a novel avenue for future vaccine development to circumvent the spreading of SARS-CoV-2.

## Supplementary information

supplemental materials

## Data Availability

All data generated and/or analyzed in this study are available on reasonable request from the corresponding author.
