# Supplementary material for: A protective AAV vaccine for SARS-CoV-2
Source: Signal Transduct Target Ther. 2022 Sep 5;7:310. doi: 10.1038/s41392-022-01158-w (PMC9443627; doi:10.1038/s41392-022-01158-w)
Supplement: Supplementary file 1 — supplemental materials [file 41392_2022_1158_MOESM1_ESM.docx]

**Supplementary Materials for**

**A protective AAV Vaccine for SARS-CoV-2**

Simeng Zhao^1,3^, Junzi Ke^1,2,3^, Bongyu Yang^1,2^, Fangzhi Tan^1^, Jie Yang^1,2^, Chao-Po Lin^2^, Haopeng Wang,^2,*^ Guisheng Zhong^1,2,*^

1. iHuman Institute, ShanghaiTech University, Shanghai 201210, China.

2. School of Life Science and Technology, ShanghaiTech University, Shanghai 201210, China.

3. These authors contributed equally.

* Wanghp@shanghaitech.edu.cn

* zhongsh@shanghaitech.edu.cn

This pdf file includes:

Materials and Methods

Figs: S1-S11

**Materials and Methods**

**Vaccine design and production**

Different domains of SARS-CoV-2 S protein were expressed under the control of the CMV promoter and terminated by an SV40 polyadenylation signal.

Research-grad and small-scale AAV vectors were homemade. Vector preparations were generated by calcium phosphate transfection of adenoviral helper plasmid, AAV-ie capsid construct, and ITR-flanked transgene plasmid in HEK293T cells. Cells and medium were harvested 96 hours after transfection. The collected cells then were treated with chloroform and the supernatant was collected. The supernatant and the medium were combined and concentrated with 1M NaCl and 10% PEG8000. After centrifugation, the pellet was resuspended in PBS buffer with DNase I (Thermo Fisher Scientific, Cat# EN0521) and RNase A (Tiangen, Cat# RT405). The crude AAVs were overlaid to iodixanol gradient solutions. After centrifugation, the AAV-containing 40% fraction was collected and concentrated. The genome-containing titers of vectors were determined by SYBR (Vazyme, Cat# Q311) analysis using primers targeting the WPRE region. The qPCR primers for WPRE are listed as follows: forward, 5’- CTTGTTTATTGCAGCTTATAATGG-3’; reverse, 5’- GATACATTGATGAGTTTGGACAAAC -3’.

***In vitro* antigen expression and detection**

Six-well plates seeded with HEK293T cells were transfected with different SARS-CoV-2 S truncated proteins expressing vectors (2µg DNA/plasmid) using the calcium phosphate transfection method. The supernatants were harvested 48 h post-transfection and mixed with reducing sample buffer (CWBio, Cat# CW0027S), heated for 8 minutes at 95°C and run on a precast 4-12% Bis-Tris PAGE gel (Genscript, Cat# M00654). Protein was transferred to a nitrocellulose (NC) filter membrane, and the membrane was blocked overnight at 4°C in PBS buffer containing 0.1% Tween 20 (PBST) and 5% non-fat milk powder. Then, the NC membrane was incubated for 1 hour in 5% milk PBST with a 1:1,000 dilution of mouse anti-His tag antibody (Proteintech, Cat# 66005-1-lg). After this, the NC membrane was washed three times with PBST buffer and subsequently incubated with 1:10,000 HRP-conjugated goat anti-mouse IgG (Proteintech, Cat# SA00001-1) in 5% milk PBST. Finally, the NC membrane was rewashed three times with PBST and imaged with ECL Western blotting detection system (Biorad, Cat# 1705062).

**Protein expression**

Recombinant SARS-CoV-2 S and RBD proteins used for ELISA binding were self-expressed and purified. The coding sequences of S-trimer ECD (residues 1-1208) or RBD were cloned into pTT5 vector with a N-terminal IL-2 signal peptide and a C-terminal 6 × His tag. For S-trimer expression, four substitutions at the cleavage site (residues 682-685) were changed to “GSAS”, two proline substitutions at the residues 986 and 987 were also introduced to stabilize the protein. The plasmids were transfected into HEK Expi293F cells (Thermo Fisher Scientific, Cat# A14527) using polyethylenimine (PEI) method. Supernatants were collected after 5 days culture, and proteins were purified with Ni-NTA resin (Qiagen, Cat# 151010181) and followed by Superose 6 10/300 gel filtration column chromatography (GE Healthcare, Cat# 17-5172-01). Proteins were then concentrated and the concentrations were determined using BCA protein concentration kit (Pierce, Cat# 23225).

**Mouse vaccination**

The mouse study was performed under the guidance of Institutional Animal Care and Use Committee (IACUC) of Shanghaitech University, China (approved number: 20210512001). Male BALB/c mice aging 6 – 8 weeks were used in this study. Mice were housed under a 12 h light/dark cycle and allowed free access to diet and water. For primary antigen screening, mice were i.v. injected with of the AAV vectored vaccines at the dose of 1 × 10^11^ GCs per mouse, after 2 weeks, the blood samples were collected from the retro-orbital plexus to prepare immune sera. For AAV-ie-S1 vaccination, the vaccine was i.m. injected at the dose of 6 × 10^10^ GCs per mouse. The blood was then collected in regular intervals to prepare immune sera. Sera were kept at -80 ^o^C before use.

**Non-human primate (NHP) vaccination**

All animal procedures were approved by the Institutional Animal Care and Use Committee of Shanghaitech University (approved number: 20210705001). *Macaca fascicularis* (2 to 5 year-old) that screened negative for viral pathogens were enrolled on the study. Animals were housed in in stainless-steel squeeze back cages, on a 12-hour timed light/dark cycle, at room temperatures. NHPs were treated with varied enrichments such as food, visual and auditory stimuli, and social interactions throughout the study. Four *Macaca fascicularis* were treated with the vaccine candidates (N = 2 males in each group) intramuscularly at a dose of 1 × 10^13^ GCs per animal (1 × 10^13^ GCs/mL, 1mL). Sera samples were obtained in regular intervals to analyze the immunogenicity of AAV-ie-S1. And whole blood samples were collected before (week 0) and after (week 8) vaccination to isolate PBMCs for cellular response analysis.

**Immunofluorescence**

Mice were i.m. or i.v. injected with AAV-ie-GFP at the dose of 1 × 10^11^ GCs per mouse. After 2 weeks, mice were anesthetized with sodium pentobarbital (40 mg/kg, i.p.) with no avoidance response to foot pinch. They were then perfused with normal saline (at 37 °C) and subsequently by ice-cold 4% PFA for fixation. Organs were post-fixed in 4% PFA for 4 h, then dehydrated in 30% sucrose. organs were frozen at −80 °C and then sectioned at 20-μm-thick with the freezing microtome (Leica CM1950) for immunofluorescence labeling. Organs sections were rinsed in PBS, permeabilized, and then blocked with blocking solution (3% w/v donkey serum and 0.5% v/v Triton X-100 in PBS) for 1.5 h at room temperature. The slices were then stained using rabbit anti-GFP antibody (Proteintech, Cat# 50430-2-AP) in 0.1% Triton X-100 and 1% serum in PBS overnight at 4 ^o^C. After washing with PBS, sections were incubated with the Alexa-488 conjugated donkey anti-rabbit antibody (Thermo Fisher Scientific, Cat# A21206) (1:1000 dilution) for 2 h at room temperature. After incubation, sections were washed with PBS. Sections were mounted with VECTASHIELD mounting medium for fluorescence with DAPI (Vectorlab, Cat# H1200). Following imaging was performed using confocal microscope (Nikon).

**Hematoxylin-eosin staining**

Fresh tissues were fixed in 4% PFA, processed, and embedded in paraffin. The tissues were sliced into sections using a rotary microtome and subjected to hematoxylin and eosin (HE) staining. The specific steps were as follows: sections were soaked in xylene for 10 minutes and repeated three times to complete dewaxing. After dewaxing, the sections were soaked in 100%, 95%, 80%, 70%, 50% and 30% ethanol for 5 minutes to dehydrated. Then, the sections were stained with hematoxylin for 8~12 minutes and rinsed with running water for 10 minutes. Put the sections into 1% hydrochloric acid ethanol differentiation liquid solution in 5~30 seconds, then rinse water for about 15 minutes. After hematoxylin staining was completed, the sections were soaked in 30%, 50%, 70%, 80% and 95% ethanol for 1 minute. Then the sections were stained in eosin solution for 3~10 seconds and soaked in 100% ethanol for 1 minute to rinse off excess eosin. Then, the sections were complete dehydrated in absolute ethanol for 5min and repeated twice. Finally, the sections were soaked in xylene for 5 minutes, repeated three twice, and sealed with neutral gum.

**ELISA**

To evaluate the antibody titers binding to SARS-CoV-2 S or RBD protein in the sera of immunized animals. Nunc Maxisorp plates (Thermo Fisher Scientific, Cat# 464718) were coated with recombinant S or RBD protein at the concentration of 2 μg/mL in PBS at 4 ^o^C overnight. After extensive washing with PBS, the plates were blocked using 5% skim milk at RT for 2 h. Serially diluted immune sera of vaccinated mice or monkeys were then added to the plates and incubated at RT for 1 h. After washes with PBS, HRP-conjugated secondary antibodies (HRP conjugated goat anti-mouse IgG: Proteintech, Cat# SA00001-1; HRP conjugated goat anti-mouse IgG2a: Proteintech, Cat# SA00012-2; HRP conjugated goat anti-mouse IgG1: Proteintech, Cat# SA00012-1; HRP-conjugated goat anti-monkey IgG: Southern Biotech, Cat# 4700-05) were added and incubated for 1 h. The plates were further washed using PBS and TMB substrate (Beyotime, Cat# P0209) was added. The absorbance at 450/620 nm was then measured with a micro plate reader (Flexstation III, Molecular devices).

**Pseudo-virus neutralization assay**

To prepare SARS-CoV-2 and related mutated pseudo-viruses, HEK-293T cells were co-transfected with pcDNA3.1-SARS-CoV-2-S or related mutants and pNL4-3.luc.RE by calcium phosphate transfection. After 72 h culture, the supernatant was collected and centrifuged at 3,000 X *g* for 10 min. To test the neutralizing activity of immune sera against pseudo-virus, HEK293T cells stably transfected with hACE2 were seeded in 96-well culture plates at a density of 5,000 cells per well. Immune sera were then diluted with pseudo-virus containing supernatant and incubated at 37 ^o^C for 1 h and then transferred to the target cells. After overnight incubation, fresh cell culture medium was changed and the cells were further cultured for two days. Luciferase activity was then analyzed using luciferase assay substrate (Promega, Cat# E1483).

**Mouse splenic cell isolation, staining, and flowcytometry**

For germinal center B (GcB) cell and follicular helper T (Tfh) cell analysis, after vaccination with AAV-ie-S1 vaccine or injection of PBS vehicle and AAV-ie-GFP vector for 9 days, mice were anesthetized with isoflurane and sacrificed. Spleens were dissected placed in cold RMPI1640 medium (Gibco, Cat# 11875119) supplemented with 10% heat inactivated fetal bovine serum (HIFBS) (Gibco, Cat# 10091148). Organs were homogenized with a syringe plunger and filtered through a 100 μm strainer on ice. Red blood cells were then lysed using Red blood lysing buffer (Beyotime, Cat# C3702). Cells were then washed with PBS and stained using LIVE/DEAD fixable aqua (Thermo Fisher Scientific, Cat# L34957) to exclude dead cells. To analyze GcB cells, cells were stained using brilliant violet 650 conjugated anti-mouse B220 (Biolegend, Cat# 103241), PE conjugated anti-mouse IgD (Biolegend, Cat# 405706), FITC conjugated anti-mouse GL7 (Biolegend, Cat# 144603), APC conjugated anti-mouse Fas (Biolegend, Cat# 152604). Cells were fixed using 4% PFA at room temperature for 15 min and then analyzed using flow cytometer (Cytoflex S, Beckman). GcB cells were defined as live B220^+^IgD^-^GL7^+^Fas^+^ cells. To analyze Tfh cells, cells stained with LIVE/DEAD fixable dye were subsequently stained using biotin labelled anti-mouse CXCR5 (eBioscience, Cat# 13-7185-82) at room temperature for 1 h. After washing with FACS staining buffer, cells were further stained with super bright 600 conjugated streptavidin (eBioscience, Cat# 63-4317-82), FITC conjugated anti-mouse CD3e (Biolegend, Cat# 100305), PE conjugated anti-mouse CD4 (Biolegend, Cat# 100511), Percp-cy5.5 conjugated anti-mouse CD44 (Biolegend, Cat# 103031). Cells were then fixed and permeabilized using Foxp3/transcription factor staining buffer set kit (eBioscience, Cat# 00-5523-00), and stained with Alexa647 conjugated anti-mouse/human Bcl6 (Biolegend, Cat# 648305) at 4 ^o^C overnight. After washing with permeabilization buffer, cells were analyzed using flow cytomete. Tfh cells were defined as live CD3^+^CD4^+^CD44^+^CXCR5^+^Bcl6^+^ cells.

For antigen-specific memory B cell (MBC) analysis, experiments were performed 14 days after vaccination. Splenic cells were prepared as described above. After stained using LIVE/DEAD fixable dye, cells were stained using biotin labelled RBD (Novoprotein, Cat# DRA43) at the concentration of 5 μg/mL for 1 h on ice. After washing with FACS staining buffer, cells were further stained with super bright 600 conjugated streptavidin, Alexa-647 conjugated streptavidin (Invitrogen, Cat# S21374). brilliant violet 650 conjugated anti-mouse B220, FITC conjugated anti-mouse IgD (Biolegend, Cat# 405703), FITC conjugated anti-mouse GL7, PE conjugated anti-mouse CD38 (Biolegend, Cat# 102708). Cells were fixed using 4% PFA at room temperature for 15 min and then analyzed using flow cytometer. RBD-specific MBCs were defined as live B220^+^IgD^-^GL7^-^ CD38^+^RBD-SB600^+^RBD-Alexa647^+^ cells.

**NHP PBMCs stimulation, intracellular staining, and flowcytometry**

The monkey whole blood samples were collected before (week 0) or after (week 8) AAV-ie-S1 vaccination, PBMCs were isolated using Ficoll (GE, Cat# 17144002) gradient centrifugation following the manufacturer’s instruction. PBMCs were cultured in RPMI 1640 medium supplemented with 10% heat inactivated fetal bovine serum (HIFBS), and penicillin/streptomycin. Cells were stimulated with synthetic SARS-CoV-2 S peptides pool (Genscript, Cat# RP30020) at the concentration of 2 μg/mL for 12 h and then incubated with 5 μg/mL Brefeldin A (MCE, Cat# HY-16592). Cells stimulated with 50 ng/mL PMA (Sigma, Cat# P8139) and 1 μM ionomycin (J&K, Cat# 464833) for 4 h were used as positive control. After stimulation, cells were washed with PBS and stained using LIVE/DEAD fixable aqua. Subsequently, cells were stained using pacific blue conjugated anti-human CD3 (BD Bioscience, Cat# 558124), FITC conjugated anti-human CD4 (BD Bioscience, Cat# 550628), PE conjugated anti-human CD8 (BD Bioscience, Cat# 557086). Intracellular staining of the cytokines was performed after fixation and permeabilization using APC conjugated anti-human IFNγ (BD Bioscience, Cat# 551385), anti-human IL2 (BD Bioscience, Cat# 551383), and anti-human TNFα (BD Bioscience, Cat# 551384) accordingly. The percentages of cytokine positive CD4^+^ or CD8^+^ cells were analyzed using flow cytometer.

**S-binding assay using FACS**

The plasmids encoding wild type SARS-CoV-2 or circulating variants (B.1.351 and B.1.1.7) S proteins with a C-terminal fused GFP were transfected into HEK293T cells. After 48 h culture, cells were dissociated and incubated with serially diluted monkey immune sera at 4 ^o^C for 1h. After PBS washing, cells were further stained using alexa-555 conjugated donkey anti-human IgG (Thermo Fisher Scientific, Cat# A21433). After extensive washing, the percentages of anti-human IgG postive cells in GFP positive cells were then analyzed using flow cytometer (Cytoflex S, Beckman). LIVE/DEAD fixable aqua (Thermo Fisher Scientific, Cat# L34957) was used to exclude dead cells.

**Statistics**

Antibody titers of ELISA assay, EC_50_ value of pseudo-virus neutralization assay were determined using non-linear regression analysis using Graphpad PRISM. Data are shown as mean ± SD. Numbers of replicates for experiments are described in the figure legends.


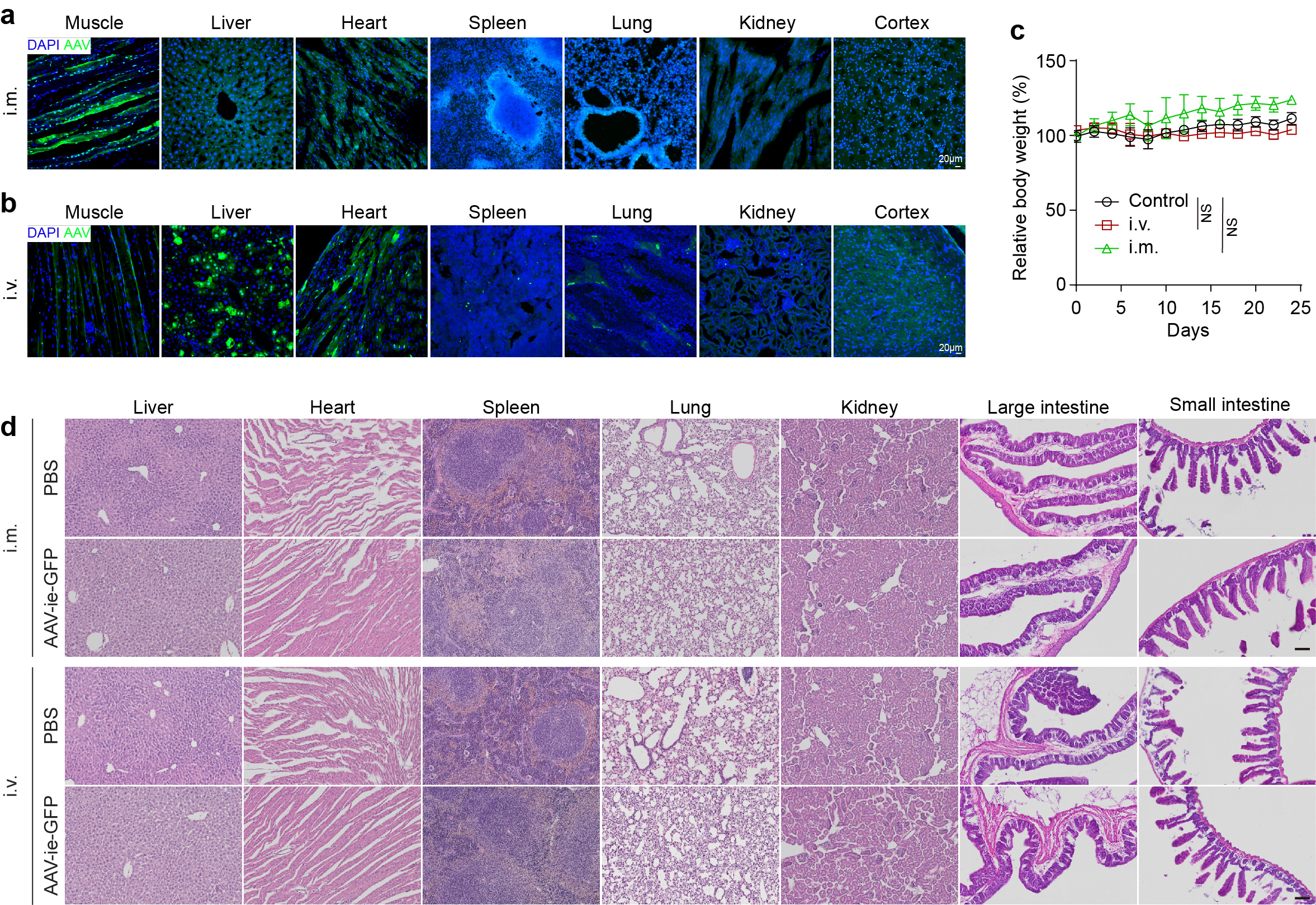


**Fig. S1. Tissue tropism of AAV-ie**. Representative fluorescence imaging of indicated organ slices after i.m. (**a**) and i.v. (**b**) injection of AAV-ie-GFP. Scale bar, 20 μm. **c**, Relative body weight of mice after i.m. or i.v. injection of AAV-ie-GFP. N = 3 in each group. **d**, Representative H&E staining images of indicated organ slices after i.m. or i.v. injection of AAV-ie-GFP and PBS control. Scale bar, 100 μm.


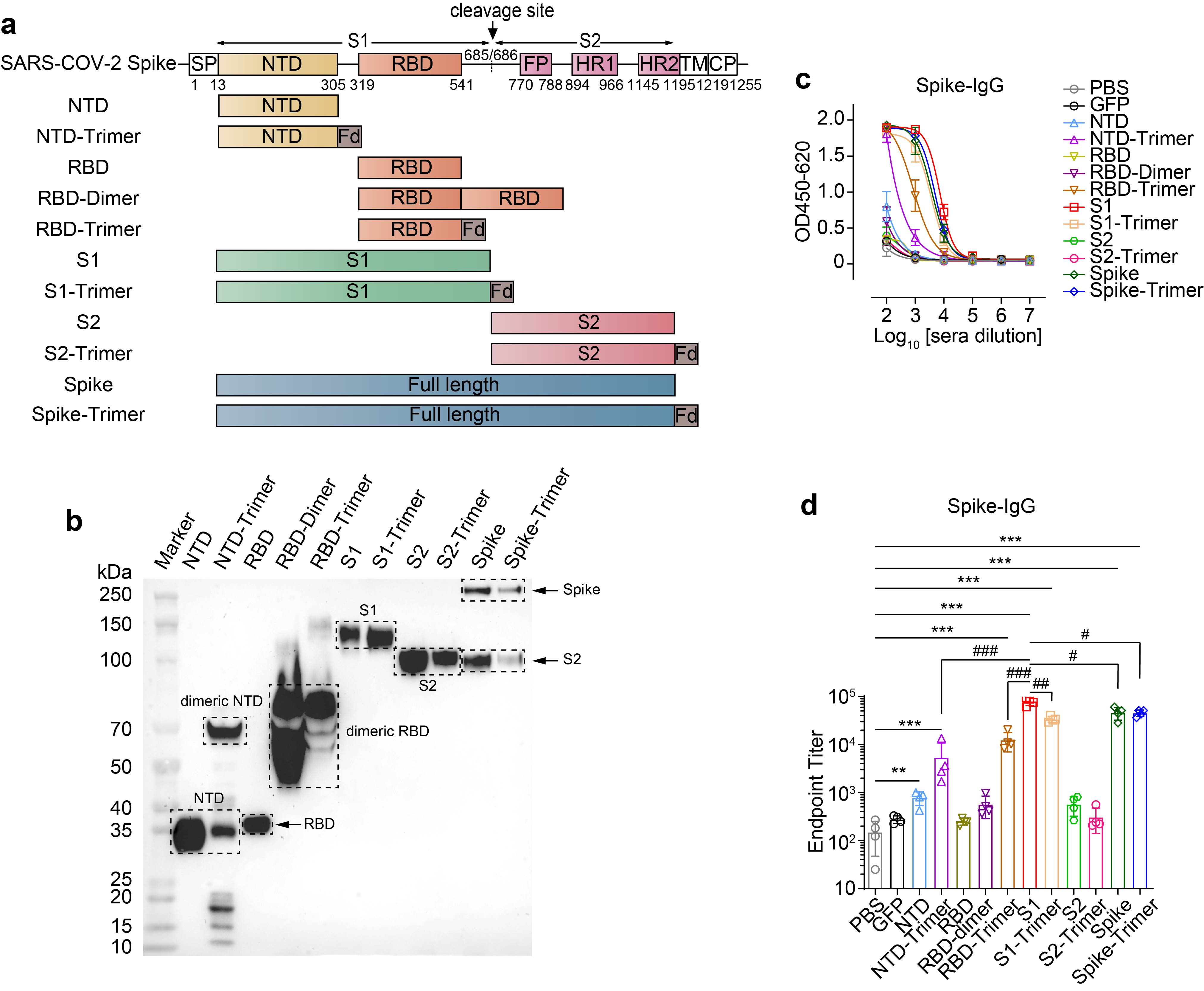


**Fig. S2. Systematic SARS-CoV-2 S antigen screening. a**, AAV-ie vectored vaccines design. **b**, Detection of S antigens expression using Western blot in HEK293T cells infected by related plasmids. **c**, S-Binding IgG antibody titers of mice sera after vaccination (i.v.) with indicated antigens at week 2, PBS and AAV-ie-GFP were used as negative control. Experiments were performed in triplicates. **d**, End-point titers summary in **c**, N = 4 in each group, ^**^ *P* < 0.01; ^***^ *P* < 0.001; ^#^ *P* < 0.05; ^##^ *P* < 0.01; ^###^ *P* < 0.001.


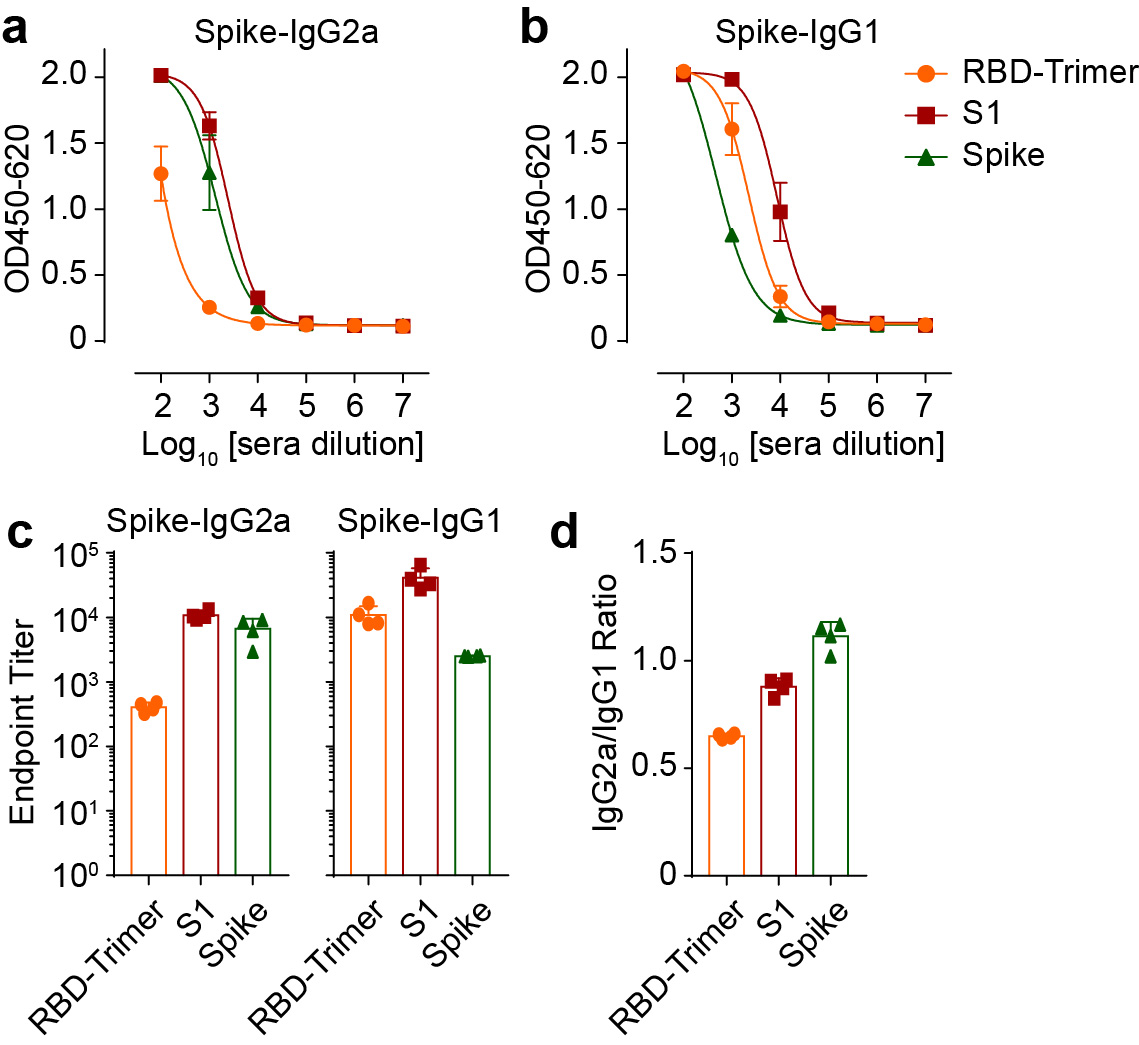


**Fig. S3. SARS-CoV-2 S-Binding antibody isotype titers of vaccinated sera in mice. a-b**, Determination of IgG2a (**a**) and IgG1 (**b**) titers using ELISA assay. Experiments were performed in triplicates. **c**, Summary of the endpoint titers of IgG2a and IgG1 in indicated immune sera (N = 4, each with 3 replicates). **d**, End-point titer ratios of IgG2a to IgG1 of three vaccines.


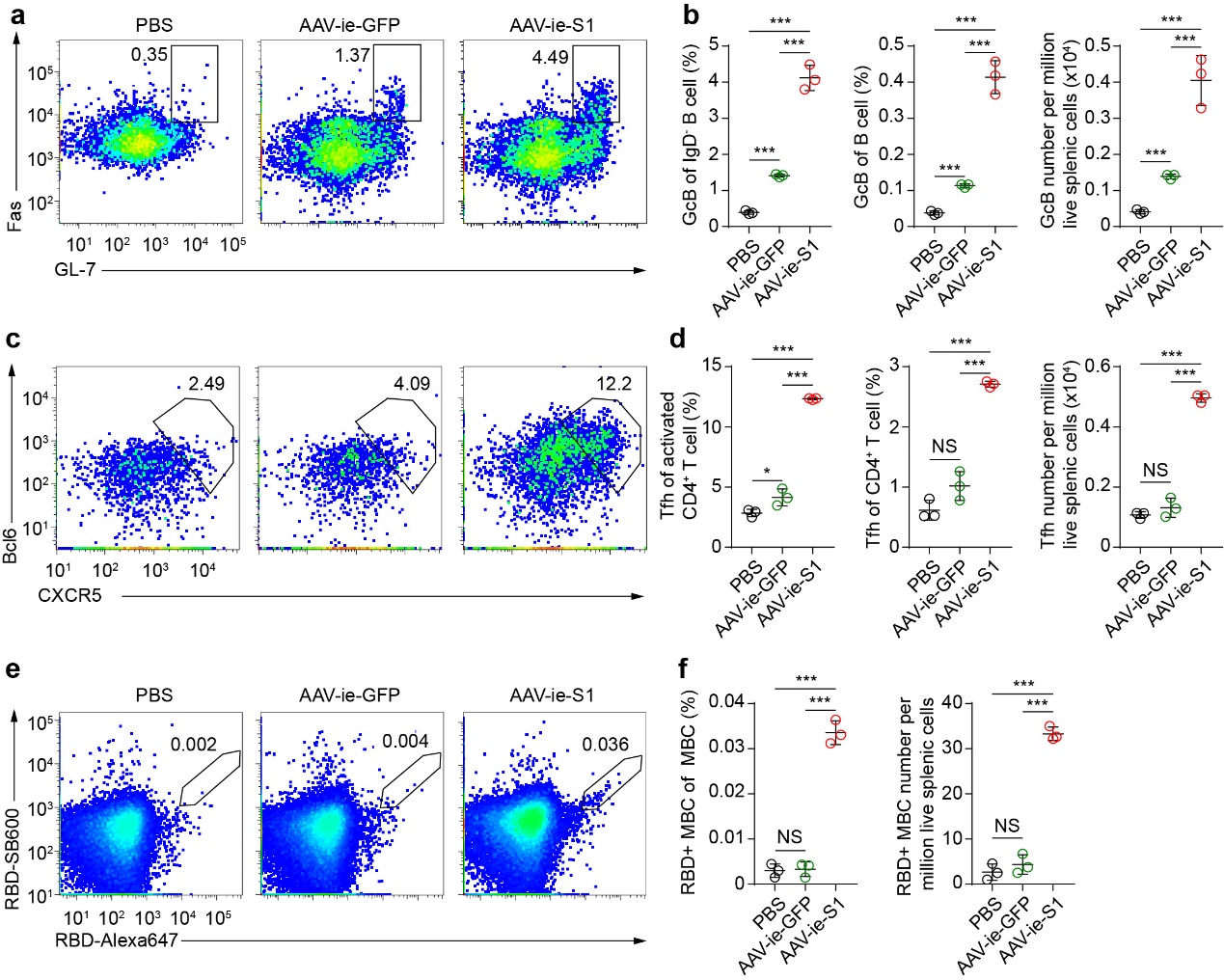


**Fig. S4. Humoral response evaluation of AAV-ie-S1 in mice. a**, Representative flow cytometry of GcB cells (defined as live B220^+^IgD^-^GL7^+^Fas^+^ cells) in spleen. **b**, Statistics of ratio and number of GcB cells in spleen. **c**, Representative flow cytometry of Tfh cells (defined as live CD3^+^CD4^+^ CD44^+^CXCR5^+^Bcl6^+^ cells) in spleen. **d**, Statistics of ratio and number of Tfh cells in spleen. **e**, Representative flow cytometry of RBD-specific memory B cells (defined as live B220^+^IgD^-^GL7^-^ CD38^+^RBD-SB600^+^RBD-Alexa647^+^ cells) in spleen. **f**, Statistics of ratio and number of RBD-specific memory B cells in spleen. N = 3 in each group, NS: not significant, ^***^ *P* < 0.001.


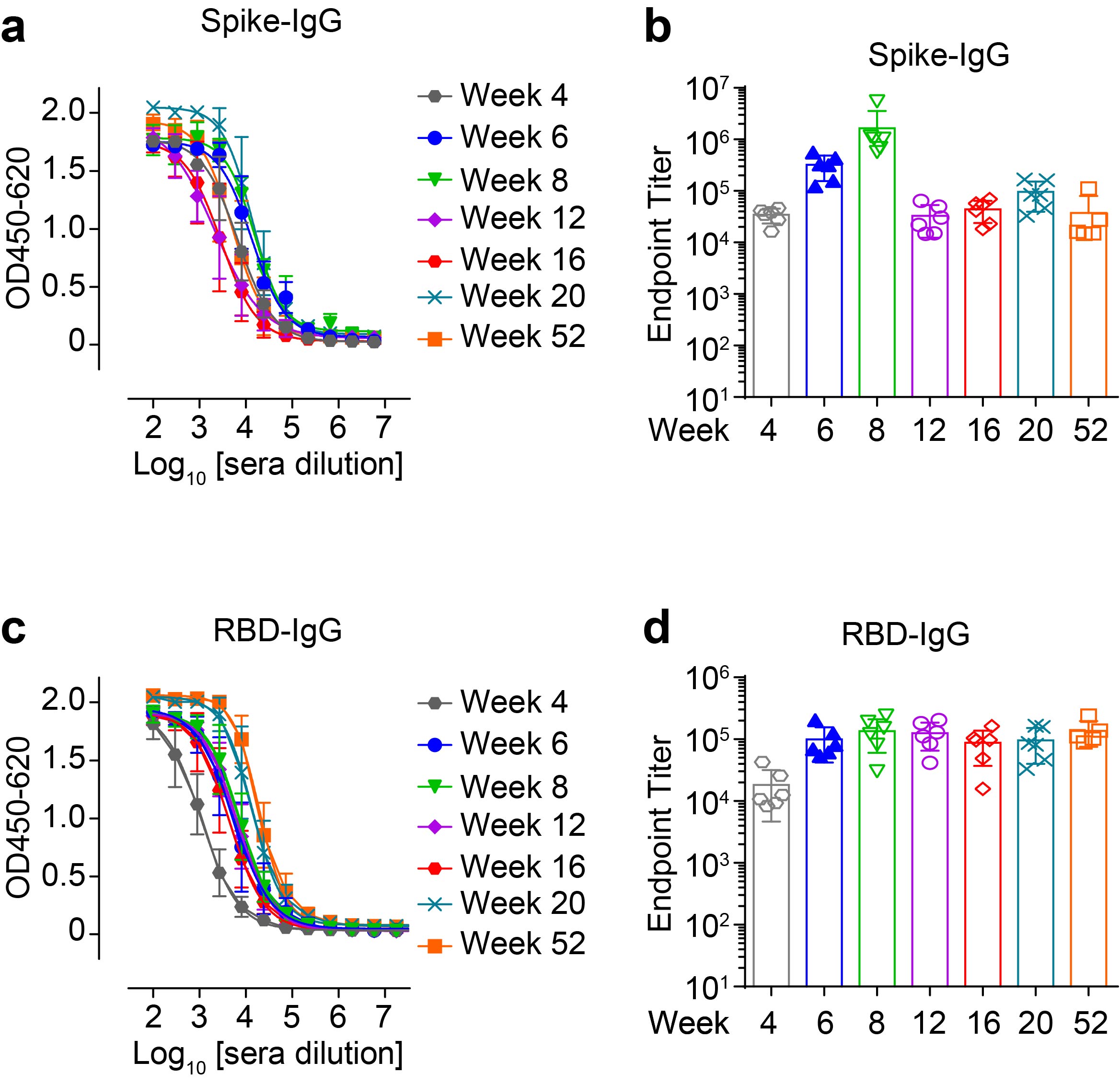


**Fig. S5. Monitoring of SARS-CoV-2 S- and RBD-Binding antibody titers of immune sera in AAV-ie-S1 vaccinated mice. a-b**, S-Binding IgG antibody titers of mice sera after vaccination (i.m.) with AAV-ie-S1 at indicated time. **a**, Determination of S-binding IgG antibody titers using ELISA assay. **b**, Endpoint titers of S-binding IgG antibody at indicated time (N = 5 - 6, each with 3 replicates). **c-d**, RBD-Binding IgG antibody titers of mice sera after vaccination (i.m.) with AAV-ie-S1 at indicated time. **c**, Determination of RBD-binding IgG antibody titers. **d**, Endpoint titers of RBD-binding IgG antibody (N = 5 - 6, each with 3 replicates).


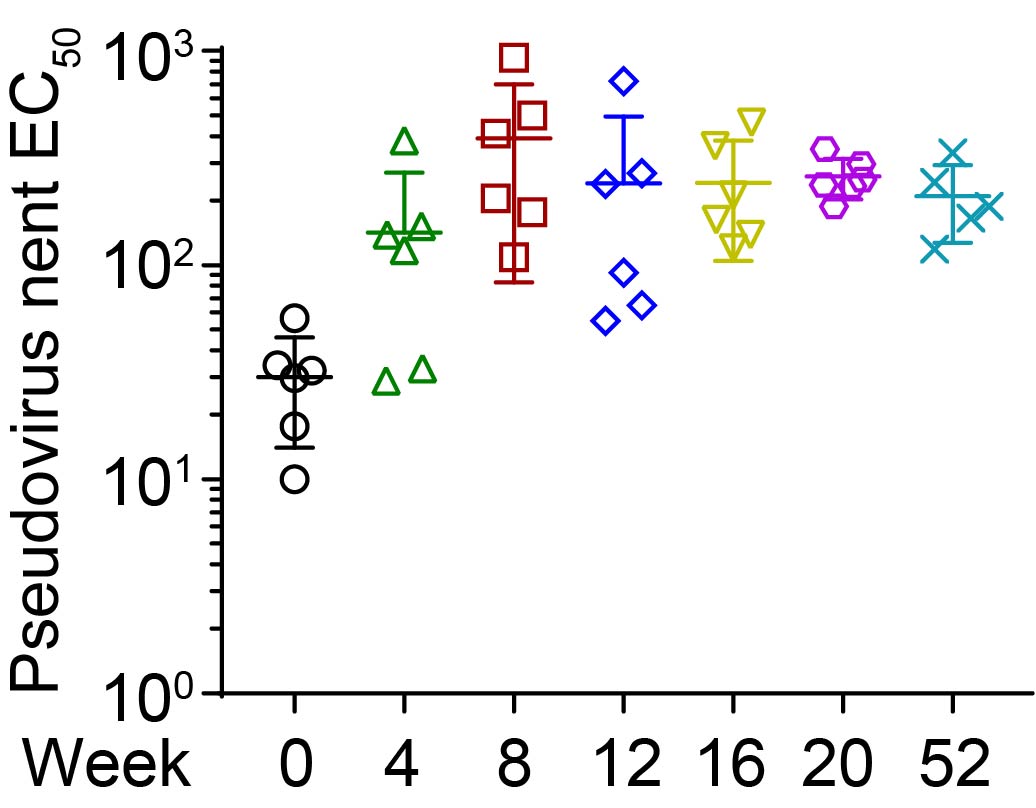


**Fig. S6. SARS-CoV-2 pseudo-virus neutralizing EC_50_ values of AAV-ie-S1 immune sera at indicated times**. Mice were vaccinated using AAV-ie-S1, and sera were then collected at indicated time for pseudo-virus neutralization. Lenti-virus based pseudo-virus particles with luciferase reporter were mixed with immune sera and then added to ACE2-expressing HEK293T cells. The luciferase signals were recorded after 48 h and the neutralizing EC_50_ values were calculated (N = 5 - 6, each with 3 replicates).


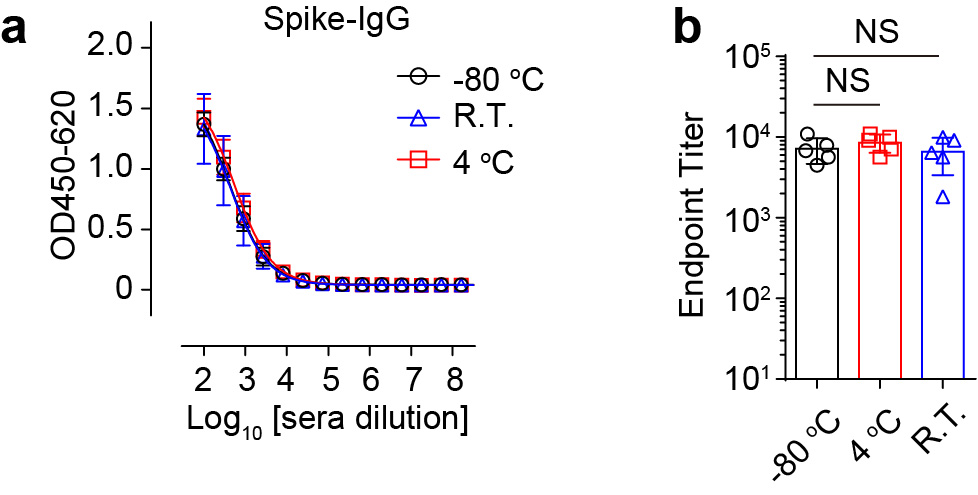


**Fig. S7. Thermostability of AAV-ie-S1**. **a**, S-Binding IgG antibody titer determination of mice sera using ELISA on week 2 after vaccination (i.m.) with AAV-ie-S1 kept at indicated temperatures for 2 weeks. Experiments were performed in triplicates. **b**, Summary of Endpoint titers. NS: no significance. (N = 5 in each group)


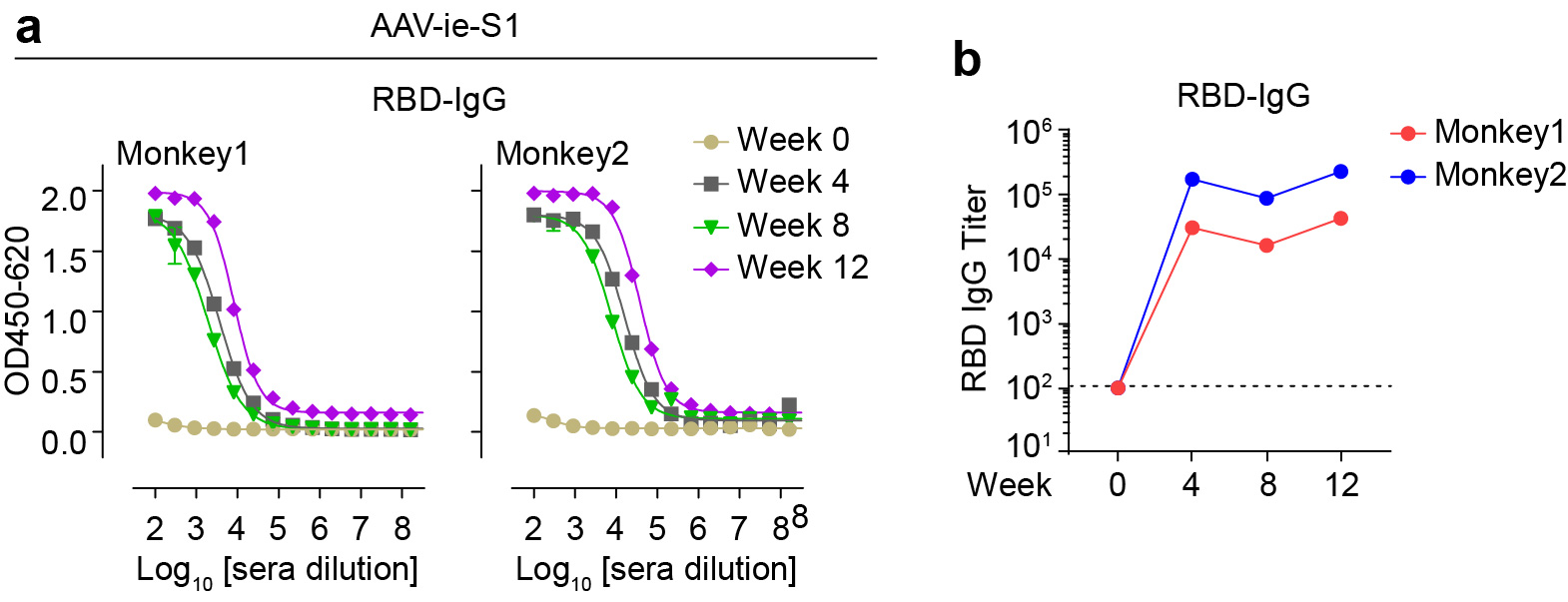


**Fig. S8. Monitoring of SARS-CoV-2 RBD-Binding antibody titers of immune sera in AAV-ie-S1 vaccinated NHPs. A**, Determination of RBD-binding IgG antibody titers using ELISA assay. Experiments were performed in triplicates. **B**, Endpoint titers of RBD-binding IgG antibody at indicated time.


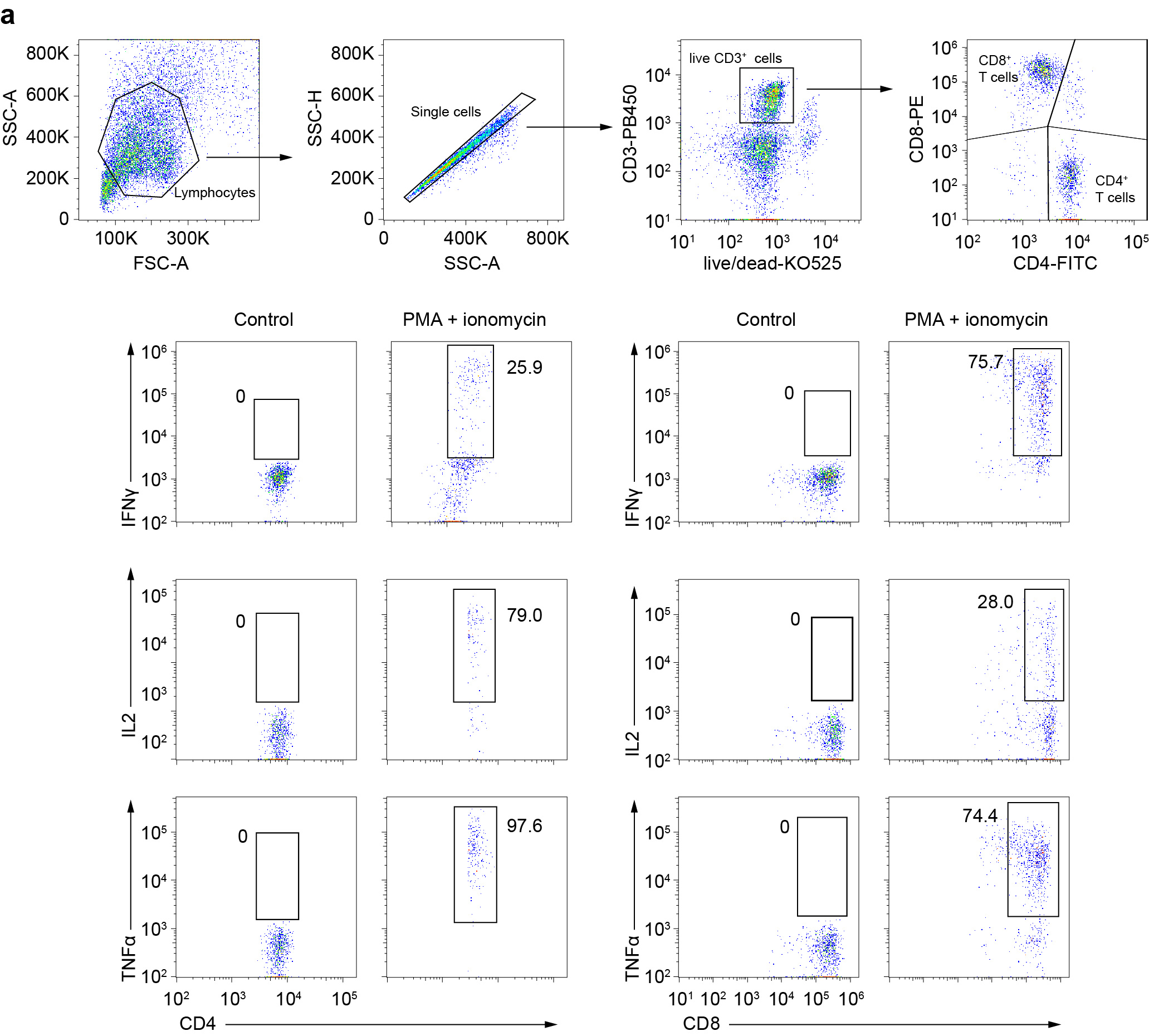

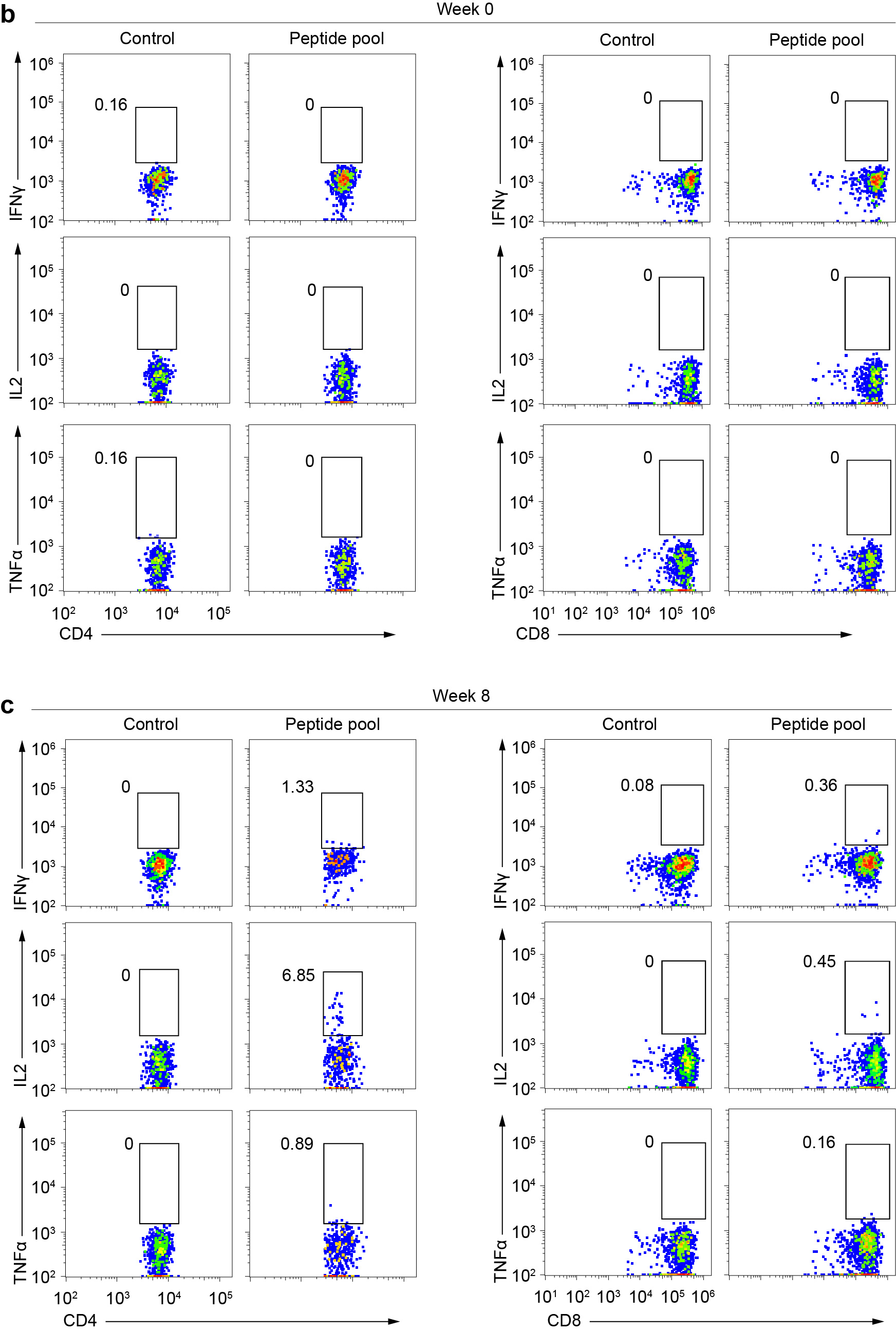


**Fig. S9. Flow cytometry analysis of SARS-CoV-2 S-specific T cells in NHPs. a**, Gating strategy. PBMCs stimulated with PMA and ionomycin were used as positive controls. **b-c**, Gating summary of SARS-CoV-2 S-specific CD4^+^ and CD8^+^ T cells before (**b**) and after (**c**) vaccination.


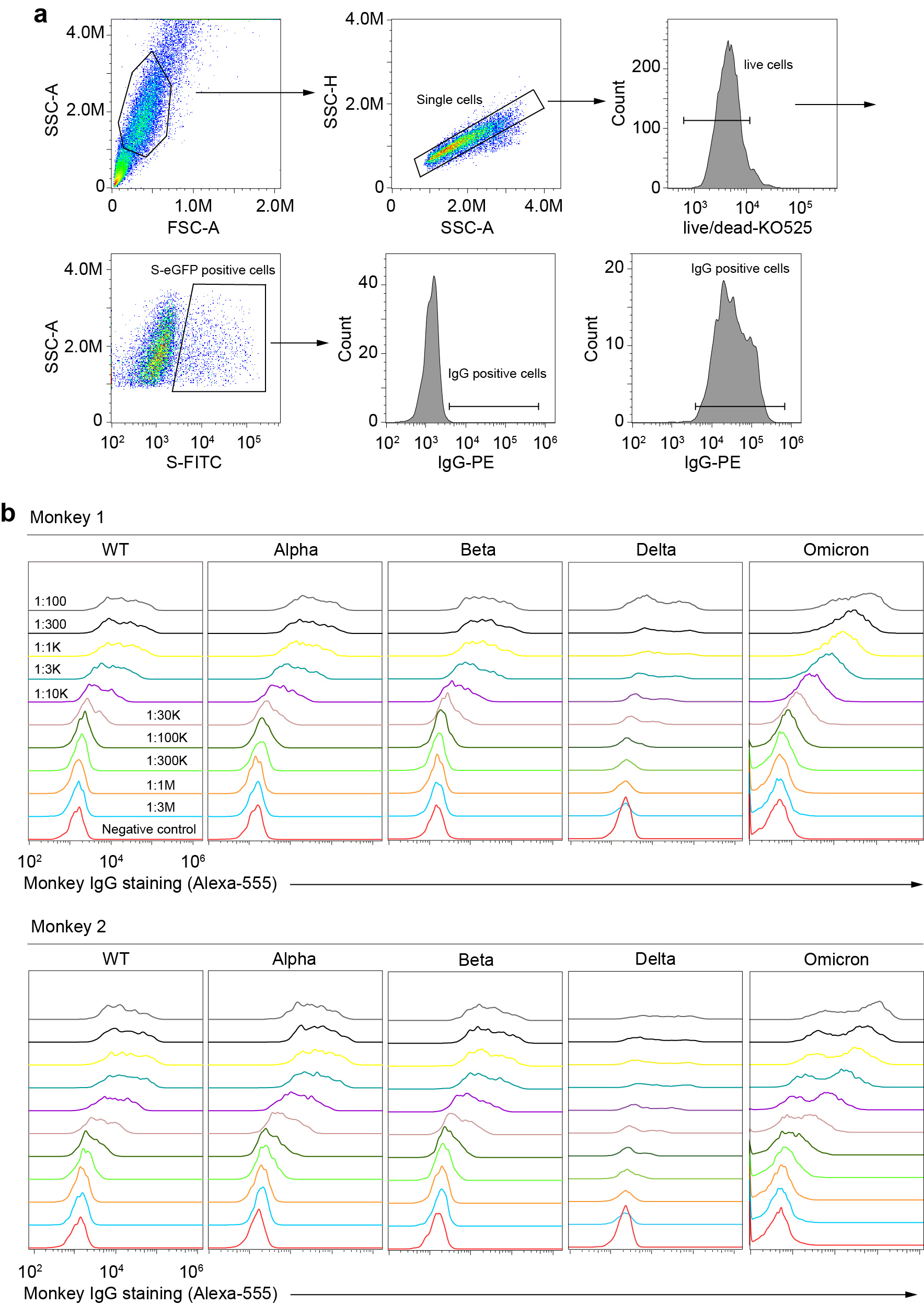


**Fig. S10. FACS-based S-binding Assay. a,** Gating strategy of FACS based SARS-CoV-2 S-Binding assay. **b**, Binding of serially diluted immune sera to S proteins of wild type, alpha, beta, and delta SARS-CoV-2 variants expressed on the surface of HEK-293T cells.


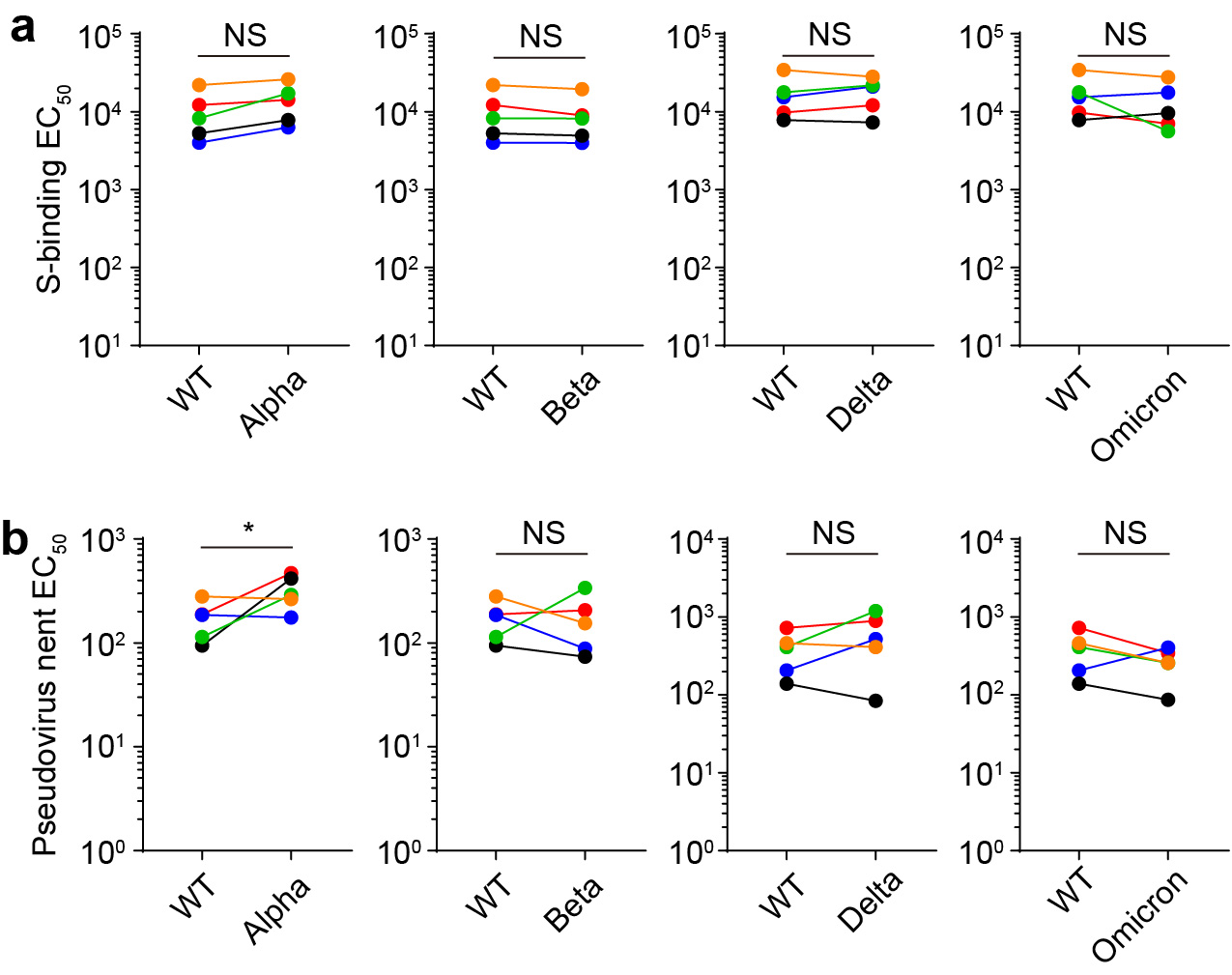


**Fig. S11. S-binding EC_50_ value change (a) and pseudovirus neutralizing EC_50_ value change (b) of mouse immune sera against alpha, beta, delta and omicron variants**. NS: not significant; ^*^ p < 0.05. N = 5 mice, experiments were performed in triplicates.
